# Antihypertensive potential of the aqueous extract which combine leaf of *Persea americana* Mill. (Lauraceae), stems and leaf of *Cymbopogon citratus* (D.C) Stapf. (Poaceae), fruits of *Citrus medical* L. (Rutaceae) as well as honey in ethanol and sucrose experimental model

**DOI:** 10.1186/1472-6882-14-507

**Published:** 2014-12-17

**Authors:** Paul Désiré Djomeni Dzeufiet, Amélie Mogueo, Danielle Claude Bilanda, Bibi-Farouck Oumarou Aboubakar, Léonard Tédong, Théophile Dimo, Pierre Kamtchouing

**Affiliations:** Department of Animal Biology and Physiology, Faculty of Science, University of Yaounde I, P.O. Box 812, Yaounde, Cameroon; Department of Physiology, High Institute of Health, “Université des Montagnes”, P.O Box 208, Bangangte, Cameroon

## Abstract

**Background:**

The present study was designed to evaluate the effects of the aqueous extract obtained from the mixture of fresh leaf of *Persea americana*, stems and fresh leaf of *Cymbopogon citratus*, fruits of *Citrus medica* and honey on ethanol and sucrose induced hypertension in rats.

**Methods:**

Rats were divided into eight groups of 6 rats each and daily treated for 5 weeks. The control group received distilled water (1 mL/kg) while rats of groups 2, 3 and 4 received ethanol 40 degrees (3 g/kg/day), 10% sucrose as drinking water and the two substances respectively. The remaining groups received in addition to sucrose and ethanol, the aqueous extract (50, 100 and 150 mg/kg) or nifedipine (10 mg/kg) respectively. Many parameters including hemodynamic, biochemical and histopathological were assessed at the end of the study.

**Results:**

The concomitant consumption of ethanol and sucrose significantly (p < 0.001) increased the blood pressure and the heart rate compared to distilled water treated-rats. The levels of total cholesterol, LDL-cholesterol, triglycerides, atherogenic index, glucose, proteins, AST, ALT, creatinin, potassium, sodium and albumin increased while the HDL-cholesterol decreased under ethanol and sucrose feeding. Chronic ethanol and sucrose intake significantly decreased the activities of superoxide dismutase (SOD) and catalase (CAT) as well as the contents of reduced glutathione (GSH) and nitrites whereas elevated the malondialdehyde (MDA) levels. Histological analysis revealed among other vascular congestion, inflammation, tubular clarification and thickening of the vessel wall in rats treated with alcohol and sucrose. Administration of the aqueous extract or nifedipine prevented the hemodynamic, biochemical, oxidative and histological impairments induced chronic ethanol and sucrose consumption.

**Conclusion:**

Current results suggest that the aqueous extract used in this study possess antihypertensive activity against ethanol and sucrose induced hypertension in rats by the improvement of biochemical and oxidative status, and by protecting liver, kidney and vascular endothelium against damages induced by chronic consumption of ethanol and sucrose.

## Background

Hypertension is currently one of the major risk factors for cardiovascular, neurological and renal events. Several studies demonstrated that excessive and chronic ingestion of ethanol causes cardiomyopathy, cardiac arrhythmias, heart failure and hypertension [1–3]. Similarly, numerous studies also indicate that diets high in carbohydrates, particularly sugars and even more particularly sucrose and fructose increase the risk of cardiovascular diseases including hypertension [4]. It has been reported that some metabolic abnormalities such as hyperinsulinemia, insulin resistance and hypertriglyceridemia as well as hyperactivity of the sympathetic nervous system and oxidative stress were frequently associated with the pathogenesis of both ethanol and sucrose induced-hypertension [5]. It is well known that hypertension can often lead to lethal complications if left untreated [6].

In spite of the large number of modern drugs, people largely use complementary and alternative medicine to prevent and cure illness [7] for curiosity and also the idea that combining it with conventional treatment would help [8]. A holistic or a spiritual health view and the belief that herbs are natural (and thus safe) also seem to be associate with the use of alternative medicine [9]. Traditionally, many of the folk remedies of plant origin have long been used for the treatment of various ailments, usually as mixture of many plants in combination with honey, palm oil or limestone [10]. Therefore, there is an urgent need to develop new and effective drugs for the treatment of hypertension. In this view, medicinal plants such as *Persea Americana*, *Cymbopogon citratus* and *Citrus medica* mixt with honey are known to have many and various metabolites possessing potential for the prevention and treatment of several diseases. *Persea americana* (avocado) is a tree belonging to the Lauraceae family which is used in traditional medicine for the treatment of ailments such as stomach ache, bronchitis, diarrhoea, diabetes and hypertension [11]. Additively, the cardioprotective effects of *Persea americana* have been widely demonstrated [12] as well as its lipid-lowering [13] and hypoglycemic effects [14]. *Cymbopogon citratus* commonly known as lemon grass is a widely used herb belonging to the Poaceae family. Phytochemical and pharmacological analysis of this plant revealed the presence of compound such as citral which acts as a calcium antagonist [15]. The plant is also known to exhibit antioxidant, anti-inflammatory, hypoglycemic, antimicrobial and chemoprotective effects [16]. Furthermore, *Cymbopogon citratus* is popularly used as antispasmodic, antipyretic, sedative, diuretic and hypotensive [17]. *Citrus medica* also called lemon is a plant belonging to the Rutaceae family. Lemons contain significant amounts of citric acid; this is why they have a low pH and a sour taste. They also contain vitamin C (ascorbic acid) which is essential to human health. These phytochemical constituents of lemon inhibit the synthesis and activity of mediators involved in inflammation such as derivatives of arachidonic acid, prostaglandins E2, F2 and thromboxane A2 [18]. The main flavonoids in the lemon are eriocitrine and hesperetin. Experiments on animals have shown that these phytochemical constituents could reduce or prevent damages related to oxidative stress [19]. Lemon through its high pectin levels is also able to dissolve fat and to lower blood cholesterol levels [20]. Honey is a natural source of energy with a relatively low glycaemic index [21]. In addition to be a source of energy, honey is an important source of antioxidants including flavonoids and phenolic compounds very effective to scavenge free radicals. These active principles contribute to reduce the risk of cardiovascular diseases and some cancers [22]. Honey is used to stimulate cell renewal (scarring), to fight tiredness and stress, to boost the immune system and possess anti-inflammatory and antibiotic activities [21]. Honey consumption also reduces the effects of ethanol [23].

Information provided by practitioners of traditional medicine in West Region of Cameroon indicates that the mixture of the fresh leaf of *Persea americana*, stems and fresh leaf of *Cymbopogon citratus*, fruits of *Citrus medica* and honey is used to treat hypertension. Therefore, the present study was undertaken to evaluate the effects of the aqueous extract resulting from that mixture on ethanol and sucrose induced hypertension in rats.

## Methods

### Animals

The antihypertensive activity of the extract was carried out on 48 males albino Wistar rats aged 6-8 weeks and weighting 150-160 g prior to the experiment. Animals were housed in standard environmental conditions under a 12/12 h light/dark natural cycle in the animal house of the Laboratory of Animal Physiology of the University of Yaounde I. All animals had free access to standard diet and tap water *ad libitum*.

### Preparation of aqueous extract

The aqueous extract was prepared from the mixture consisted of fresh leaf of *Persea Americana* (70 g), fresh leaf and stems of *Cymbopogon citratus* (110 g), fruits of *Citrus medica* (300 g) and honey (500 g). These biological materials were harvested in Bandjoun in the West Region of Cameroon in March 2012 and identified at the National Herbarium by comparison to the voucher specimen N° 57756 NHC for *Persea Americana*, N° 16628/SFR/Cam for *Cymbopogon citratus* and N° 65106/HNC for *Citrus medica*. The mixture mentioned above was boiled in 2.5 L of distilled water for 40 minutes. The resulting crude aqueous extract was filtered using a filter paper (Whatmann N° 3) followed by evaporation at 40°C using an oven (Memmert, Germany). The yield of the extraction was 24.31%.

### Ethical consideration

All animal treatment procedures used in the present study were approved by the Cameroon National Ethical Committee (Ref. N° FWIRB 00001954).

### Experimental design

The antihypertensive activity of the aqueous extract was evaluated by using ethanol and sucrose induced hypertension in rats as previously described [2]. Rats were randomly divided in eight groups of six rats each and daily treated for five consecutive weeks.

Animals of the first group (control) received by oral gavage distilled water (1 mL/kg/day). Rats of group 2 were treated by oral gavage with ethanol 40° (3 g/kg/day), group 3 received sucrose 10% as drinking water and group 4 received simultaneously ethanol 40° and sucrose 10%. Rats of groups 5-8 received by oral gavage in addition to ethanol and sucrose, the aqueous extract (50, 100 and 150 mg/kg) or the calcium inhibitor, nifedipine (10 mg/kg) respectively. During the experimental period, the body weight was assessed twice a week. At the end of the investigation period, blood pressure and heart rate of all rats were measured according to the method previously described [2]. Briefly, each rat was anesthetized using an intraperitoneal injection of urethane (1.5 g/kg). The trachea was exposed and cannulated to facilitate spontaneous breathing. The arterial blood pressure and heart rate were measured from right carotid artery via an arterial cannula connected to a pressure transducer coupled with a hemodynamic recorder Biopac Student Lab. (MP35) and computer. Thirty minutes of equilibration period were observed before each measure. Changes in mean arterial pressure were calculated using the equation below:


### Biochemical and histological analysis

After recording hemodynamic parameters, rats were sacrificed and the free running blood was collected and centrifuged at 3000 rpm for 15 minutes. The serum obtained was stored at -20°C for the determination of some biochemical markers. Serum samples were assayed for triglycerides, total cholesterol, HDL-cholesterol, albumin, creatinin, alanine aminotransferase (ALT) and aspartate aminotransferase (AST) using the commercial diagnostic kit Fortress. The atherogenic index (AI) was calculated by the following formula: AI = ([total cholesterol] – [HDL-cholesterol)])/ [HDL-cholesterol)] [24]. The glucose assay was performed using the kit Inmesco while sodium ions were quantified using the Atlas Medical kit.

After blood collection, organs (aorta, liver and kidney) of each animal were dissected out and part of each organ was homogenized. Aorta was homogenized in 10 volumes of Mc Even solution whereas liver and kidney were homogenized in 5 volumes of 50 mM Tris-HCl. The homogenate (20% for aorta and 10% for the other organs) were centrifuged at 3000 rpm for 15 minutes at 4°C. The supernatant collected was stored at -20°C for further biochemical studies. The total protein was performed by the method of Gornall et al. [25] and the activity of superoxide dismutase (SOD) was assessed according to the protocol described by Misra and Fridovish [26]. Reduced glutathione (GSH) and catalase were assayed following the method described by Ellman [27] and Sinha [28] respectively. Nitrites content was estimated using the protocol of Slack [29] while the concentration of malondialdehyde was determined by the method described by Wilbur et al. [30]. For microscopic evaluation, part of investigated organs was fixed in 10% formalin for 7 days and paraffin embedded for microscopical examination in accordance with routine laboratory procedure. Paraffin sections of 4 μm were prepared and stained with haematoxylin and eosin (H&E) and Masson Trichrome for histological examination. Morphometric measurements of the thickness of arteries were performed using the axio HOME system (Carl Zeiss, Oberkochen, Germany). Briefly, the system consists of an IBM-PC compatible computer using the 2.04 version of the Zeiss-Alcatel TITN Answare software (Meylan, France) and an in-built light microscope. Six arteries per animal were quantified, given a total of 36 vessels per group.

### Statistical analysis

Statistical analysis was performed using SPSS 16.0. All data were analysed by one-way analysis of variance (ANOVA) followed by the Tukey post hoc test and expressed as mean ± standard error of the mean (SEM). The difference was considered significant at p < 0.05.

## Results

### Effects of the extract on body weight, blood pressure and heart rate

Changes in body weight of the control and treated rats are presented in Table 1. Rats gained weight during the experimental period with no significant difference in weight gain at the end of the study between controls and rats treated with the aqueous extract at the doses of 50, 100 and 150 mg/kg.

**Table 1:** **Effects of the extract on body weight gain, blood pressure and heart rate**

	Body weight gain (g)	DAP (mm Hg)	MAP (mm Hg)	SAP (mm Hg)	HR (BPM)
**Water**	76.20 ± 1.98	76.92 ± 1.40	90.46 ± 1.29	117.50 ± 1.27	352.60 ± 3.77
**Ethanol**	79.95 ± 3.68	96.90 ± 1.38^aα^	114.50 ± 1.67^aα^	149.70 ± 2.46^aα^	398.40 ± 2.06^aα^
**Sucrose**	59.17 ± 1.91	98.48 ± 1 .40^aα^	116.50 ± 1.78^aα^	152.72 ± 2.67^aα^	392.00 ± 3.52^aα^
**EtOH+ Su**	52.02 ± 1.79	109.08 ± 1.29^a^	129.70 ± 0.99^a^	171.12 ± 2.71 ^a^	423.80 ± 2.37^a^
**EtOH + Su + Nif**	51.42 ± 2.31	90.68 ± 1.54^aα^	106.00 ± 1.41^aα^	136.70 ± 1.37^aα^	372.60 ± 2.30^aα^
**EtOH + Su + Ext50**	67.57 ± 2.62	80.92 ± 1.36^α^	94.44 ± 1.39^α^	121.48 ± 1.66^α^	360.80 ± 1.95^α^
**EtOH + Su + Ext100**	59.40 ± 2.03	77.50 ± 1.14^α^	89.84 ± 1.05^α^	114.54 ± 1.9^α^	350.60 ± 1.88^α^
**EtOH + Su + Ext150**	60.02 ± 1.53	76.94 ± 1.55^α^	88.81 ± 1.52^α^	112.56 ± 1.74^α^	352.80 ± 3.01^α^

Each value represents a mean ± S.E.M.; n = 5; ^a^p < 0.001: significantly different compared to water. ^α^p < 0.001: significantly different compared to ethanol and sucrose. DAP = diastolic arterial blood pressure, MAP = mean arterial blood pressure, SAP = systolic arterial blood pressure, HR = heart rate, EtOH + Su = Ethanol and Sucrose; Ext50, Ext100 and Ext150 = Extract at the respective doses of 50, 100 and 150 mg/kg; Nif = Nifedipine (10 mg/kg).

Variations in systolic arterial blood pressure (SAP), mean arterial blood pressure (MAP), diastolic arterial blood pressure (DAP) and heart rate (HR) are presented in Table 1. Chronic consumption of ethanol 40° (3 g/kg/day) and/or sucrose 10% for 5 weeks significantly increased (p <0.001) arterial blood pressure and heart rate as compared with rats receiving distilled water. The increase of MAP and HR was by 25.97% and 12.98% in ethanol-treated group, by 28.02% and 11.17% in sucrose-fed group and by 41.80% and 22.74% in group receiving simultaneously the two substances. The increase of blood pressure and heart rate induced by ethanol and sucrose consumption was significantly (p <0.001) prevented in groups that received additionally the aqueous extract or nifedipine. The MAP and HR of rats that were orally administered the extract were lowered respectively by 16.86% and 4.94% at 50 mg/kg, by 25.81% and 7.95% at 100 mg/kg and by 28.95% and 10.56% at 150 mg/kg compared to ethanol and sucrose treated group. Similarly, the administration of nifedipine (10 mg/kg) decreased by 29.46% and 10.00% the MAP and HR respectively compared with the group submitted to ethanol and sucrose.

### Effects of the extract on some serum biochemical parameters

#### Effects on lipid profile and glucose content

As shown in Table 2, the daily ingestion of ethanol 40° (3 g/kg) or sucrose (10%) *ad libitum* for 5 consecutive weeks significantly increased by 34.43% and 42.30% the total cholesterol, by 40.00% and 45.41% the content of triglycerides and by 52.39% and 56.18% the atherogenic index while the HDL-cholesterol decreased by 38.66% and 40.11% respectively compared to normal control group. In group treated simultaneously with ethanol and sucrose, the significant increase of the level of total cholesterol, triglycerides and atherogenic index was respectively 49.84%, 68.75% and 65.30% whereas the HDL-cholesterol decrease was 50.96% compared to distilled water-treated rats. Treatment with the aqueous extract (50, 100 and 150 mg/kg) in combination with ethanol and sucrose prevented the variation of serum total cholesterol, triglycerides, atherogenic index and HDL-cholesterol as compared to the ethanol-sucrose-treated group. At the higher dose (150 mg/kg), the aqueous extract significantly reduced the total cholesterol by 42.23%, triglycerides by 80.00% and the atherogenic index by 44.49% meanwhile the HDL-cholesterol increased by 89.01% when compared to ethanol and sucrose treated group. Nifedipine (10 mg/kg) administered in addition to ethanol and sucrose significantly improved lipid profile as compared to untreated-rats.

**Table 2:** **Effect of the extract on blood glucose and lipid profile**

	Glucose (mg/dL)	Triglycerides (mg/dL)	Total cholesterol (mg/dL)	HDL–cholesterol (mg/dL)	Atherogenic index
**Water**	110.60 ± 10.65	152.38 ± 9.98	156.41 ± 8.61	74.49 ± 5.43	0.51 ± 0.07
**Ethanol**	152.50 ± 13.29^c^	213.33 ± 7.33^aα^	210.25 ± 15.74	45.68 ± 4.40^a^	0.78 ± 0.03^b^
**Sucrose**	165.95 ± 8.23^c^	221.58 ± 4.53^aβ^	222.56 ± 17.16^c^	44.61 ± 4.31^a^	0.80 ± 0.02^b^
**EtOH+ Su**	153.31 ± 10.42^c^	257.14 ± 2.24^a^	234.35 ± 15.16^b^	36.52 ± 3.18^a^	0.84 ± 0.01^a^
**EtOH + Su + Nif**	113.76 ± 13.25	51.42 ± 5.25^aα^	153.84 ± 6.01 ^β^	70.59 ± 4.61^α^	0.54 ± 0.04^α^
**EtOH + Su + Ext50**	126.27 ± 13.47	90.79 ± 4.77^aα^	185.12 ± 14.80	47.00 ± 3.57^b^	0.74 ± 0.01^b^
**EtOH + Su + Ext100**	101.48 ± 8.53^γ^	68.57 ± 4.77^aα^	145.12 ± 14.47^β^	55.38 ± 3.00^γ^	0.61 ± 0.02^γ^
**EtOH + Su + Ext150**	110.11 ± 9.59^γ^	51.42 ± 3.38^aα^	135.38 ± 9.84^α^	69.04 ± 3.94^α^	0.47 ± 0.08^α^

Each value represents a mean ± S.E.M.; n = 5; ^a^p < 0.001; ^b^p < 0.01, ^c^p < 0.05: significantly different compared to water. ^α^p < 0.001, ^β^p < 0.01, ^γ^p < 0.05: significantly different compared to ethanol and sucrose. EtOH + Su = Ethanol and Sucrose; Ext50, Ext100 and Ext150 = Extract at the respective doses of 50, 100 and 150 mg/kg; Nif = Nifedipine.

The serum level of glucose was 110.60 ± 10.65 mg/dL in normal control group (Table 2). The value of this biochemical parameter significantly (p <0.05) increased by 37.87% in ethanol-treated group, by 50.04% in sucrose-fed group and by 38.61% in ethanol-sucrose-fed group compared to distilled water-treated group. However, in group treated with the aqueous extract in addition to ethanol and sucrose, the serum glucose level significantly (p <0.05) decreased by 33.80% at 100 mg/kg and by 28.18% at 150 mg/kg compared to untreated rats. In the same condition, nifedipine (10 mg/kg) lowered by 25.79% the serum glucose content compared to rats simultaneously treated with ethanol and sucrose.

#### Effects on some parameters of the liver and kidney functions

Repeated oral ingestion of ethanol, sucrose or the two ingredients for 5 weeks increased by 59.00%, 72.80% and 85.86% the serum total protein and by 30.02%, 33.85% and 29.06% the albumin content (Table 3). Furthermore, chronic ethanol and/or sucrose feeding promoted significant increase in AST and ALT activities compared to control group. In ethanol-sucrose treated group, AST and ALT activities increased by 111.81% and 96.20% respectively compared to distilled water-treated group. However, the administration of the aqueous extract (50, 100 and 150 mg/kg) or nifedipine (10 mg/kg) in association with simultaneous ethanol and sucrose intake resulted in a significant decrease in serum total proteins, albumin, AST and ALT when compared to ethanol-sucrose untreated rats. At the dose of 150 mg/kg, the extract significantly (p < 0.001) lowered by 85.25% the total proteins content and by 50.64% and 56.12% the activity of AST and ALT respectively compared with ethanol-sucrose-treated group.

**Table 3:** **Effect of the extract on some biochemical parameters related to liver and kidney function**

	ALT (UI/L)	AST (UI/L)	Protein (mg/mL)	Albumin (mg/dL)	Creatinin (mg/dL)	Potassium (mmol/L)	Sodium (mEq/L)
**Water**	26.77 ± 3.84	70.96 ± 10.20	23.53 ± 1.51	26.08 ± 2.52	0.38 ± 0.06	6.30 ± 0.79	136.41 ± 5.51
**Ethanol**	46.44 ± 5.24	116.12 ± 16.69	37.42 ± 5.17^c^	33.91 ± 3.41	0.96 ± 0.07^a^	9.89 ± 0.93	169.80 ± 7.32
**Sucrose**	45.42 ± 5.65	130.96 ± 11.65^c^	40.66 ± 1.64^b^	34.91 ± 3.18	0.90 ± 0.03^a^	9.43 ± 0.99	149.80 ± 10.49
**EtOH+ Su**	52.54 ± 4.79^c^	150.32 ± 11.10^a^	43.74 ± 4.21^a^	33.66 ± 3.88	1.07 ± 0.04^a^	10.44 ± 0.71^c^	166.01 ± 7.99
**EtOH + Su + Nif**	22.71 ± 3.15^β^	65.16 ± 7.59^α^	5.72 ± 1.02^bα^	23.24 ± 1.88	0.48 ± 0.05^α^	5.10 ± 0.76^α^	122.08 ± 10.75^γ^
**EtOH + Su + Ext50**	34.91 ± 6.72	115.48 ± 10.06	16.81 ± 2.52^α^	32.29 ± 1.70	0.83 ± 0.06^a^	9.26 ± 0.56	139.00 ± 9.49
**EtOH + Su + Ext100**	27.79 ± 3.28^γ^	92.25 ± 12.60^γ^	6.73 ± 1.25^bα^	28.26 ± 1.67	0.77 ± 0.06^bγ^	7.48 ± 0.81^γ^	123.03 ± 10.82^γ^
**EtOH + Su + Ext150**	23.05 ± 3.65^β^	74.19 ± 12.45^β^	6.45 ± 0.67^bα^	26.24 ± 1.02	0.52 ± 0.06^α^	5.70 ± 0.96^β^	117.46 ± 10.80^γ^

Each value represents a mean ± S.E.M.; n = 5; ^a^p < 0.001; ^b^p < 0.01, ^c^p < 0.05: significantly different compared to water. ^α^p < 0.001, ^β^p < 0.01, ^γ^p < 0.05: significantly different compared to ethanol and sucrose. EtOH + Su = Ethanol and Sucrose; Ext50, Ext100 and Ext150 = Extract at the respective doses of 50, 100 and 150 mg/kg; Nif = Nifedipine (10 mg/kg).

As summarized in Table 3, chronic ethanol and/or sucrose consumption affected the kidney function in the current study by increasing significantly serum creatinin, potassium and sodium levels compared to control rats treated with distilled water. In group receiving simultaneously ethanol and sucrose during 5 weeks of experimental period, the increase in the content of these kidney markers was 182.45% (p < 0.001) for creatinin, 65.64% (p < 0.01) for potassium and 21.69% (p < 0.05) for sodium. As compared to ethanol-sucrose treated group, the aqueous extract (100 and 150 mg/kg) reduced the serum creatinin by 27.95% (p < 0.05) and 50.93% (p < 0.001), potassium by 28.33% (p < 0.05) and 45.38% (p <0.01) and sodium by 25.89% (p <0.05) and 29.25% (p < 0.05) respectively. Nifedipine administered in the same condition significantly prevented the rise in serum creatinin, potassium and sodium levels induced by ethanol and sucrose.

### Effects of the extract on some parameters of oxidative stress

#### Effects of the extract on total protein

**Figure 1:**
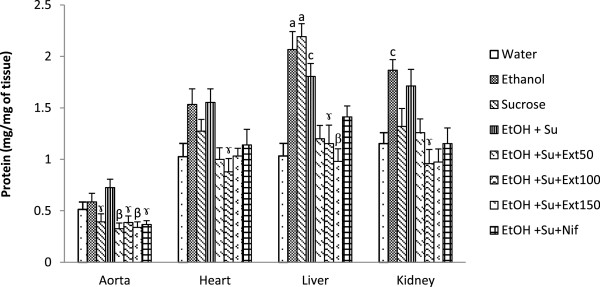
**Effect of the extract on tissue proteins.** Each bar represents a mean ± S.E.M.; n = 5; ^a^p < 0.001, ^c^p < 0.05: significantly different compared to water. ^β^p < 0.01, ^γ^p < 0.05: significantly different compared to ethanol and sucrose. EtOH + Su = Ethanol and Sucrose; Ext50, Ext100 and Ext150 = Extract at the respective doses of 50, 100 and 150 mg/kg; Nif = Nifedipine.

As shown in Figure 1, the total protein content in the liver was increased by 112.25% (p < 0.05) in group treated with sucrose (10%) and by 74.83% (p < 0.05) in ethanol and sucrose administered rats as compared with control. The association of the extract or nifedipine in the treatment of rats submitted to the ingestion of ethanol and sucrose induced a reduction of total protein in all investigated tissues as compared with untreated rats.

#### Effects of the extract on reduced glutathione content

**Figure 2:**
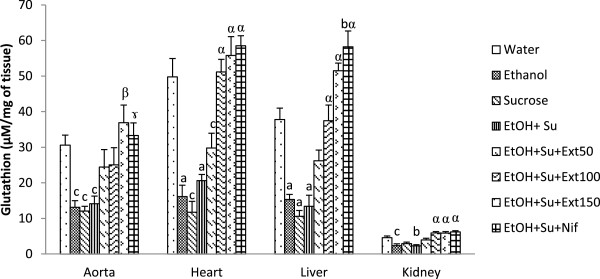
**Effect of the extract on reduced glutathione levels.** Each bar represents a mean ± S.E.M.; n = 5; ^a^p < 0.001; ^b^p < 0.01, ^c^p < 0.05: significantly different compared to water. ^α^p < 0.001, ^β^p < 0.01, ^γ^p < 0.05 : significantly different compared to ethanol and sucrose. EtOH + Su = Ethanol and Sucrose; Ext50, Ext100 and Ext150 = Extract at the respective doses of 50, 100 and 150 mg/kg; Nif = Nifedipine.

Figure 2 shows the concentration of reduced glutathione (GSH) in tissues of normal and experimental groups. GSH content was found to be decreased in ethanol and/or sucrose fed-animals in comparison with distilled-water treated rats. The decrease of this parameter in rats treated simultaneously with ethanol and sucrose was 53.49% (p < 0.05) in aorta, 58.63% (p < 0.001) in heart, 64.46% (p < 0.001) in liver and 50.24% (p < 0.01) in kidney as compared to normal control value. Oral administration of the aqueous extract or nifedipine significantly restored the amount of GSH in both rat tissues when compared to ethanol-sucrose-treated group. The increase in the content of this non-enzymatic antioxidant in group receiving the extract at 150 mg/kg was 161.25% (p < 0.01) in aorta, 170.94% (p < 0.001) in heart, 283.04% (p < 0.001) in liver and 157.91% (p < 0.001) in kidney.

#### Effects of the extract on superoxide dismutase and catalase activities

**Figure 3:**
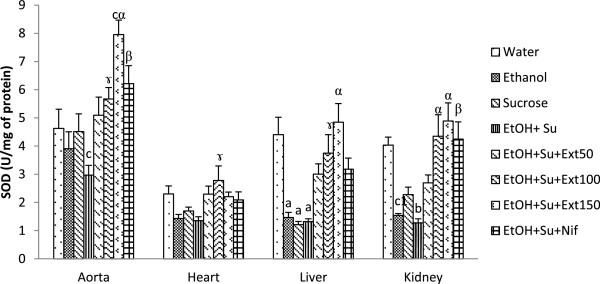
**Effects of the extract on the activities of superoxide dismutase.** Each bar represents a mean ± S.E.M.; n = 5; ^a^p < 0.001; ^b^p < 0.01, ^c^p < 0.05 : significantly different compared to water. ^α^p < 0.001, ^β^p < 0.01, ^γ^p < 0.05: significantly different compared to Ethanol and sucrose. EtOH + Su = Ethanol and Sucrose; Ext50, Ext100 and Ext150 = Extract at the respective doses of 50, 100 and 150 mg/kg; Nif = Nifedipine.

**Figure 4:**
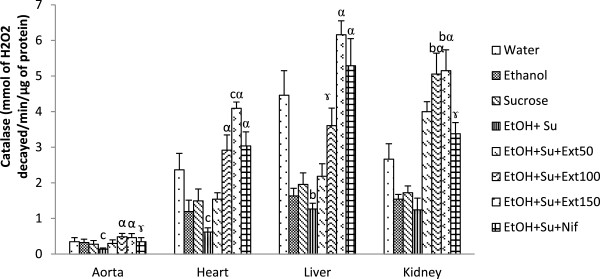
**Effects of the extract on the activities of catalase.** Each bar represents a mean ± S.E.M.; n = 5; ^b^p < 0.01, ^c^p < 0.05: significantly different compared to water. ^α^p < 0.001, ^γ^p < 0.05: significantly different compared to ethanol and sucrose. EtOH + Su = Ethanol and Sucrose; Ext50, Ext100 and Ext150 = Extract at the respective doses of 50, 100 and 150 mg/kg; Nif = Nifedipine.

The activities of antioxidant enzymes superoxide dismutase (SOD) and catalase in homogenates tissues of control and experimental rats are illustrated by Figures 3 and 4. A significant depletion of SOD activity in liver and heart were observed following ethanol and/or sucrose treatment. In group simultaneously treated with ethanol and sucrose, the activity of SOD was lowered by 35.84% (p < 0.05) in aorta, by 69.81% (p < 0.001) in liver and by 68.36% (p < 0.01) in kidney compared with normal control rats. Interestingly, the activity of catalase was significantly decreased only in the liver when rats were treated either with ethanol or sucrose, but in group receiving both substances, this activity was found to be significantly reduced in all tested tissues. Oral administration of the extract (100 and 150 mg/kg) exhibited significant increase in the activity of the enzymatic markers of oxidative status mentioned above compared with ethanol-sucrose untreated group. At the dose of 150 mg/kg, the extract improved respectively the activity of catalase and SOD by 238.98% (p < 0.001) and 167.87% (p < 0.001) in aorta, by 559.25% (p < 0.001) and 61.07% (p > 0.05) in heart, by 387.27% (p < 0.001) and 264.46% (p < 0.001) in liver and by 314.81% (p < 0.001) and 282.90% (p < 0.001) in kidney compared with untreated group.

#### Effects of the extract on malondialdehyde content

**Figure 5:**
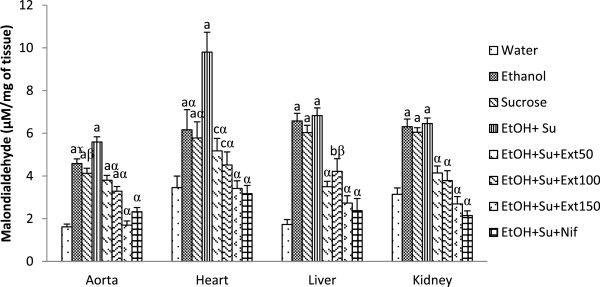
**Effects of the extract on malondialdehyde content.** Each bar represents a mean ± S.E.M.; n = 5; ^a^p < 0.001; ^b^p < 0.01, ^c^p < 0.05: significantly different compared to water. ^α^p < 0.001, ^β^p < 0.01, ^γ^p < 0.05: significantly different compared to ethanol and sucrose. EtOH + Su = Ethanol and Sucrose; Ext50, Ext100 and Ext150 = Extract at the respective doses of 50, 100 and 150 mg/kg; Nif = Nifedipine.

Figure 5 shows that ethanol and sucrose alone or together significantly (p < 0.01) increased the level of the end product of lipid peroxidation, malondialdehyde (MDA) in various investigated tissues when compared with normal control rats. The MDA content increased by 247.20% in aorta, by 184.05% in heart, by 294.79% in liver and by 105.41% in kidney in ethanol-sucrose ingested rats as compared with distilled water-fed group. Treatment with the doses of 50, 100 and 150 mg/kg of the aqueous extract as well as nifedipine (10 mg/kg) significantly (p < 0.001) prevented the increase of MDA as compared to ethanol-sucrose-administered rats.

#### Effects of the extract on nitrites content

**Figure 6:**
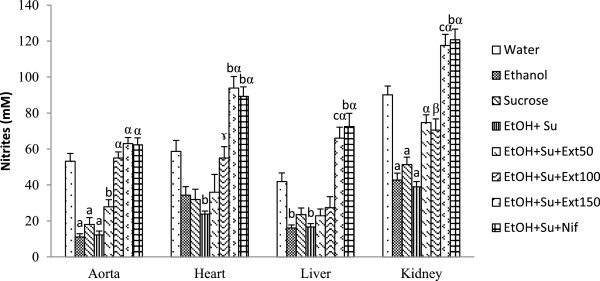
**Effects of the extract on nitrites content.** Each bar represents a mean ± S.E.M.; n = 5; ^a^p < 0.001; ^b^p < 0.01, ^c^p < 0.05: significantly different compared to water. ^α^p < 0.001, ^β^p < 0.01: significantly different compared to ethanol and sucrose. EtOH + Su = Ethanol and Sucrose; Ext50, Ext100 and Ext150 = Extract at the respective doses of 50, 100 and 150 mg/kg; Nif = Nifedipine.

Figure 6 represents the level of nitrites in aorta, heart, liver and kidney of different rat groups. Results of this study clearly revealed that ethanol and sucrose intake alone or together decrease the concentration of nitrites in the above tissues compared with normal control group. As compared to distilled water-fed rats, nitrites content in group daily treated with ethanol and sucrose declined by 76.98% (p < 0.001) in aorta, by 59.39% (p < 0.01) in heart, by 60.21% (p < 0.01) in liver and by 56.79% (p < 0.001) in kidney. Treatment with the aqueous extract (100 and 150 mg/kg) as well as nifedipine (10 mg/kg) prevented the decline of nitrites content compared to ethanol-sucrose untreated rats. The level of nitrites in rats treated with the extract (100 mg/kg) was increased by 349.09% (p < 0.001) in aorta, by 130.84% (p < 0.05) in heart, by 64.00% (p > 0.05) in liver and by 81.14% (p < 0.01) in kidney when compared with ethanol and sucrose untreated group.

#### Effects of the extract on some histological parameters

**Figure 7:**
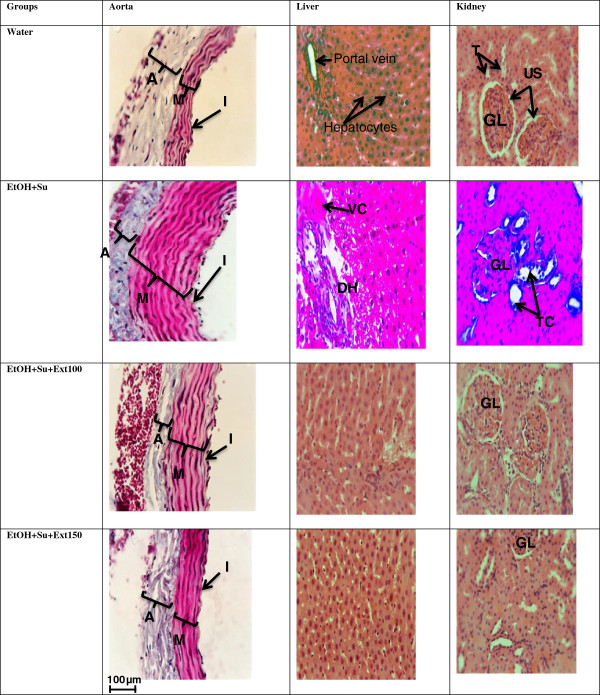
**Micro photography of aorta liver and kidney.** Haematoxylin/Eosine and Masson Trichrome staining; X400; n = 36 for aorta; EtOH + Su = Ethanol and Sucrose; Ext50, Ext100 and Ext150 = Extract at the respective doses of 50, 100 and 150 mg/kg. A: Adventice; I: Intima; M: Media; CV: Vascular Congestion; GL: Glomeruli; TC: Tubular Clarification; HD: hepatocytes Degeneration; US: urinary space; T: Tubules.

Hematoxylin and eosin stained section showed normal aspect in the morphology of aorta, liver and kidney of control group treated with distilled water (Figure 7). The daily ingestion of ethanol and sucrose for 5 weeks produced vascular congestion, hepatocytes degeneration and dilatation of sinusoids capillaries in the liver. Similarly, ethanol and sucrose induced tubular clarifications in the kidney and thickness in the arterial walls leading to the reduction in the blood vessel diameter. The thickness of the aorta of ethanol-sucrose untreated rats (376.31 ± 15.31 μm) significantly (p < 0.001) increased by 159.31% when compared to normal rats. The aqueous extract (100 and 150 mg/kg) prevented the morphological modifications induced by ethanol and sucrose in investigated organs. As compared to untreated rats, the thickness of aorta in rats treated with the extract (100 and 150 mg/kg) was reduced (p < 0.001) by 36.45 and 60.05% respectively.

## Discussion

In the present study, ethanol-sucrose induced hypertension was used to assess the antihypertensive effects of the aqueous extract obtained from the mixture of fresh leaf of *Persea americana*, stems and fresh leaf of *Cymbopogon citratus*, fruits of *Citrus medica* and honey. Results obtained showed that chronic high consumption of ethanol and/or sucrose increase blood pressure and heart rate. These findings are in agreement with previous studies demonstrating that ethanol and/or sucrose can cause hypertension, one component of a multifacetal metabolic-hemodynamic complex that also includes dyslipidemia, enhanced vascular resistance and oxidative stress [2, 3]. Results disclose that the aqueous extract is efficient as antihypertensive agent by significantly and dose-dependently preventing the increase of blood pressure and heart rate in ethanol-sucrose hypertensive rats. Indeed, various findings have shown the presence of phytocompounds such as flavonoids, alkaloids, tannin and phenolic compounds in the different medicinal plants and honey used to prepare the aqueous extract [11, 19, 22, 31]. The antihypertensive effects of this extract may be due to the presence of the above phytochemical components which are known for their vasorelexant and cardioprotective activities [32].

Dyslipidemia and hyperglycemia are significant and independent risk factors of cardio vascular disease. Ethanol-sucrose-treated rats showed a significant decreased of HDL-cholesterol level and increased the levels of total cholesterol, triglycerides and LDL-cholesterol as well as atherogenic index. These findings are in agreement with results obtained by Bilanda et al. [2] who reported that hypercholesterolemia and dyslipidemia are associated with the pathogenesis of hypertension induced by chronic ethanol and sucrose intake. Administration of the aqueous extract dose-dependently provided a beneficial action on rat lipid profile with regard to reduction of total cholesterol, triglycerides and LDL-cholesterol and the increase of HDL-cholesterol. The lipid lowering potential of the extract may be attributed to the presence of phytochemical constituents like flavonoids, saponins and tannins [13, 19, 22]. Flavonoids are reported to lower LDL-cholesterol and increase HDL-cholesterol concentrations in hypercholesteremic animals [33]. Saponins are reported to inhibit pancreatic lipase activity in high fat diet fed mice leading to greater fat excretion due to reduced intestinal absorption of dietary fats [34]. Similarly, tannins are recognized for their ability to inhibit lipid absorption [35]. The aqueous extract also markedly reduced atherogenic index which is considered to be a better indicator of coronary heart disease risk than individual lipoprotein concentration [36]. Moreover, the extract prevented the increase in the serum glucose level induced by chronic ethanol and sucrose feeding suggesting its hypoglycaemic activity. The improvement of blood glucose level induced by the aqueous extract was associated with a reduction of serum triglycerides and total cholesterol as previously mentioned for most hypoglycaemic treatments [37]. The glucose lowering effect of the extract may be due to its ability to improve the function of pancreatic β cells by increasing cells sensitivity to insulin and/or the rate of glucose transport and utilization [14].

The present investigation revealed that chronic ethanol and sucrose consumption produced significant increase in ALT and AST activities, two enzymes reported to be sensitive indicator of liver injury [38]. Generally, any damage of the parenchymal liver cells results in elevation of both transaminases in the blood and AST found in the serum is of both mitochondrial and cytoplasmic origin and any rise can be taken as a first sign of cell damage that leads to the outflow of the enzymes into the serum [39]. Increased in serum transaminases in the present study was also accompanied by the elevation in serum total protein and histological abnormalities such as vascular congestion, sinusoid dilatation and inflammation in the liver of ethanol-sucrose hypertensive rats. Furthermore, we also observed an increase in the serum creatinin content in groups treated with ethanol and/or sucrose when compared with normal control group. Indeed, the serum creatinin levels is known as a reliable and sensitive indicator of renal function and any rise in this biochemical parameter is observed if there is marked damage to functional nephrons [40]. In animals simultaneously treated with ethanol and sucrose, it has been also observed a significant increase in serum Na^+^ and K^+^. This result is in agreement with previous studies of Kang et al. [32] who showed that hypernatremia and hyperkalemia were associated with the pathogenesis of fructose induced hypertension in rats. The increase in these biochemical markers of renal function in ethanol-sucrose hypertensive group was confirmed by histological analysis which revealed tubular clarification. Treatment with the aqueous extract reversed the increase of those biochemical indicators and histological changes induced by ethanol and sucrose intoxication in the liver and the kidney. This potential activity of the extract might be owing to the presence of secondary metabolites such as flavonoids, polyphenols and alkaloids [41].

The present investigation showed that ethanol and sucrose feeding reduced SOD and catalase activities as well as the GSH content. Decreased activity of superoxide dismutase in chronic ethanol and sucrose exposure may be due to excessive generation of superoxide anion leading to the inactivation of this enzyme meanwhile, declined activity of catalase may be due to the loss of NADPH, generation of superoxide, increased activity of lipid peroxidation or combination of all [42]. A major function of GSH is detoxification of xenobiotics and/or their metabolites. The depletion of GSH content in ethanol and sucrose-treated rats may be due to its oxidation by the reactive oxygen intermediates generated during the metabolism of ethanol and inhibition of the metabolic synthesis and increased rates of turnover [43]. Our study has shown that SOD and catalase activities as well as the GSH content were restored following the extract administration, suggesting that the efficacy of this extract may be partially attributed to its capacity to improve oxidative status. Malondialdehyde (MDA) is one of the end-products of polyunsaturated fatty acid peroxidation and is a good indicator of the degree of lipid peroxidation [44]. Our results indicated increase in MDA level in the homogenates tissues of ethanol and/or sucrose-fed animals, suggesting enhanced lipid peroxidation leading to tissue damage and failure of antioxidant defence mechanisms to prevent the formation of excessive free radicals [38]. The reduced MDA content upon administration of the extract point out the favourable impact of this extract in breaking the chain reaction of lipid peroxidation engendered by chronic ethanol and sucrose ingestion. Indeed, the different plants and honey used to prepare our aqueous extract are reported to contain various bioactive compounds such as flavonoids, alkaloids, tannin and polyphenols which are known for their antioxidant activities. These compounds are able to capture free radicals and thus protect the cell membrane or cell integrity [32, 45, 46].

The vasorelaxant function of the endothelium is in great part mediated by the production of nitric oxide (NO). Current findings showed that the nitrites content significantly decreased in investigated tissues of rats treated with ethanol and sucrose compared to their normal control. The above result may be due to unfavourable outcome of superoxide anion generated during ethanol and sucrose feeding which can avidly reduce nitric oxide by transferring its extra electron to NO in order to form peroxynitrite, a potent oxidant which can antagonize the vasodilatory effects of NO [47]. Treatment with the aqueous extract reversed the adverse effect of prolonged ethanol and sucrose intake on nitrites content suggesting its ability to improve the endothelial function in this animal model of secondary hypertension. This potential may be due to the presence of secondary metabolites like flavonoid, alkaloids and phenols which are able to stimulate the production of nitric oxide and to scavenge superoxide anion [48].

## Conclusion

In conclusion, this study demonstrates the potential of the aqueous extract prepared by mixing the fresh leaf of *Persea americana*, stems and fresh leaf of *Cymbopogon citratus*, fruits of *Citrus medica* and honey to prevent ethanol and sucrose-induced hypertension in rats. Moreover, the extract improved biochemical parameters of lipids profile, liver and kidney functions as well as oxidative status. Thus, this study provides scientific validation of the empirical use of this medicine in the management of hypertension and could serve as a basis of the formulation of improved traditional drug for hypertension. However, more study need to be carried out in order to determine the exact mechanism of action of our extract.
